# Relationship between single nucleotide polymorphisms in the 3′UTR of amyloid precursor protein and risk of Alzheimer’s disease and its mechanism

**DOI:** 10.1042/BSR20182485

**Published:** 2019-05-02

**Authors:** Qiong Zhou, Lian Luo, Xiaohang Wang, Xiang Li

**Affiliations:** 1 Department of Neurology, Ningbo First Hospital, 59 Liuting Road, Ningbo, Zhejiang, China; 2 Department of Neurology, Xixi Hospital of Hangzhou, Hengbu Street 2, Xihu District, Hangzhou, Zhejiang, China; 3 Department of Neurology, First Affiliated Hospital of Zhejiang Chinese Medical University, 54 Youdian Road, Hangzhou, Zhejiang, China; 4 Department of Neurology, The First Affiliated Hospital of Wenzhou Medical University, No.2 Fuxue Lane, Wenzhou, Zhejiang, China

**Keywords:** Alzheimer’s disease, amyloid precursor protein, microRNA, single nucleotide polymorphism

## Abstract

**Background and objective:** Deregulation of the expression of amyloid precursor protein (APP) can lead to the development of Alzheimer’s disease (AD). Recent studies have shown that many single nucleotide polymorphisms (SNPs) in the 3′ untranslated region (UTR) of APP are associated with the development of AD. Since microRNAs (miRNAs) are involved in the regulation of APP expression, we believe that the APP 3′UTR polymorphism may affect the regulation of APP expression in miRNAs. **Results**: The levels of miR-101-3p, miR-153-3p, miR-144-3p, miR-381-3p, and miR-383-5p in plasma of patients with AD were significantly lower than those in the control group. The *APP*-534G/A site A allele was a protective factor for AD risk (adjusted odds ratio (OR) = 0.700, 95% confidence interval (95% CI): 0.573–0.840, *P*<0.001). The *APP*-369C/G site variation was not associated with AD risk. The *APP*-118C/A site A allele was a protective factor for AD (adjusted OR = 0.762, 95% CI: 0.639–0.897, *P*=0.001). The *APP*-534G/A site mutation affects the regulation of APP protein expression by miR-101-3p, miR-144-3p, miR-153-3p, and miR-381-3p, and the mutation of the *APP*-118C/A site affects miR-101-3p, miR-144-3p, miR-153-3p, and miR-383-5p regulation of APP expression. **Conclusion:** APP 3′UTR polymorphisms can affect the regulation of APP expression by miRNAs and thus affect the occurrence of AD.

Alzheimer’s disease (AD) is a common geriatric disease whose incidence is increasing worldwide [1]. Current treatment can only delay the progress of cognitive impairment, which makes the early diagnosis of AD particularly important. Pathologically, AD is caused by the accumulation of aggregated and hyperphosphorylated τ protein in cells and the extracellular deposition of amyloid-β (Aβ) peptides derived from the hydrolysis of amyloid precursor protein (APP) [2]. Studies have shown that mutations of *APP* can lead to abnormal APP processing and accumulation, to the deposition of abnormal Aβ, and eventually lead to neuronal death [3]. There are also related studies showing that elevated APP protein levels can lead to abnormal elevation of Aβ production, inducing neurodegeneration and dementia [4–6].

MicroRNAs (miRNAs) are a class of small non-coding RNAs that regulate gene expression after gene transcription and are negative regulators of gene expression regulation, thereby playing an important role in neuronal function and survival [7,8]. miRNAs interact with the 3′ untranslated region (UTR) of a target messenger RNA (mRNA) transcript through a partially complementary form, resulting in mRNA destabilization and/or translational inhibition [9,10]. The expression level of APP protein is closely related to the occurrence of AD. TargetScan (http://www.targetscan.org/mamm_31/) predicts that the targets of APP protein, including miR-101-3p, miR-144-3p, miR-153-3p, and miR-381-3p, are all APP 3′UTR. Vilardo et al. [11] showed that miR-101-3p is a negative regulator of APP expression and affects the accumulation of amyloid β, suggesting that miR-101-3p may play a role in neuropathological disorders. Liang et al. [12] identified the binding site of miR-153-3p on APP 3′UTR by luciferase assay, and confirmed that miR-153-3p inhibited APP expression by experiments. Overexpression of miR-144-3p is ubiquitous in primate brain and AD patients, and miR-144-3p acts as a negative regulator of a disintegrin and metalloprotease 10 (ADAM10) in the pathogenesis of AD [13]. MiR-185 is capable of targeting DNA methyltransferase 1 and modulating DNA methylation in human gliomas [14], but it is not clear whether it can regulate APP expression. MiR-381-3p regulates stromal cell-derived factor-1 (SDF-1) SDF-1/CXC chemokine receptor type 4 (CXCR-4) CXCR4 signaling pathway through leucine-rich to repair of nerve damage in acute cerebral ischemia after cerebral lymphatic obstruction [15]. However, whether miR-381-3p is involved in the regulation of APP expression is currently lacking evidence. MiR-383-5p may play a key role in focal cerebral ischemia by regulating post-transcriptional levels of peroxisome proliferator-activated receptor γ (PPARγ) expression [16], whether it has function during the pathogenesis of AD is not clear.

There is increasing evidence that genetic variation in sites that bind to miRNAs may significantly increase the risk of neurodegenerative diseases. For example, Wang et al. [17] showed that the single nucleotide polymorphisms (SNPs) of the 3′UTR of the fibroblast growth factor 20 (*FGF20*) gene may affect its binding to miR-433 and lead to an increased risk of PD [18]. The role of miRNAs in the development of AD has been supported by a large body of research, and a variety of miRNAs have also been used as markers for the diagnosis of AD [19–22]. Simultaneously, a number of AD-specific gene mutations have been identified in the 3′UTR of APP and BACE1, for example, Brouwers et al. [23] showed that APP-369C>G and -534G>A increased the APP promoter activity by nearly two-fold. Theuns et al. [24] identified three mutations (-118C→A, -369C→G and -534G→A) in European patients with AD, showing a nearly two-fold increase in neuronal specificity of APP transcriptional activity *in vitro*.

In the present study, we analyzed whether the expression of APP is regulated by miRNAs such as miR-101-3p, miR-144-3p, miR-153-3p, and miR-381-3p in a Chinese Han patient population with AD, and whether the expression regulation was related to the polymorphisms at the APP 3′UTR-534G/A, -369C/G, and -118C/A loci.

## Materials and methods

### General data

A total of 385 patients with AD treated in our hospital from March 2015 to January 2018 were enrolled in the present study, including 200 males and 185 females aged 60–85 years with a mean age of 73.4 ± 8.9 years. All AD patients were sporadic. The diagnostic criteria for AD were based on the diagnostic criteria for neurological communication disorders and strokes, the Association of Alzheimer’s Disease and Related Diseases (NINCDS-ADRDA) [25], and the Alzheimer’s Disease Diagnostic Practice Standard (OCDAD). Exclusion criteria were: a diagnosis of cognitive impairment caused by vascular dementia, Lewy body dementia, frontotemporal dementia, Parkinson’s disease dementia, and other diseases excluding AD [26]. For the control group, 385 physical examinees without AD were recruited and matched to the age and sex of the patients; these participants all had a Mini-Mental State Examination (MMSE) score higher than 24, and the group consisted of 201 males and 184 females, aged 62–81, with an average age of 72.5 ± 7.8 years. All subjects in the study signed informed consent and complied with the Declaration of Helsinki.

### Cell culture

Human HeLa cells were cultured in Dulbecco’s modified Eagle’s medium (DMEM) supplemented with 10% heat-inactivated fetal bovine serum (Invitrogen, Carlsbad, CA, U.S.A.). One day before transfection, HEK293 cells were inoculated into a 24-well plate with 1.0 × 10^5^ cells per well, and HeLa cells were inoculated into a six-well plate with 20% confluency. According to the manufacturer’s instructions, Lipofectamine 2000 (Invitrogen) was used for transfection.

### Construction of each genotype plasmid of APP 3′UTR-534G/A, -369C/G, and -118C/A loci

Mutagenesis of APP 3′UTR -534G/A, -369C/G, -118C/A loci were carried out by TOPgene Technology (Montreal, Quebec, Canada) and verified by sequencing.

### Plasma miRNA level detection

A volume of 5 ml blood was extracted from all subjects for genomic DNA extraction. According to the manufacturer’s instructions, miRNAs were extracted from isolated plasma using miRNeasy Serum/Plasma Kit (Qiagen GmbH, Hilden, Germany). The miRNAs were analyzed using quantitative real-time polymerase chain reaction (qRT-PCR).First, miRNA reverse transcription kits (Applied Biosystems, Foster City, CA, U.S.A.) with miRNA primers were used to reverse transcribed miRNA into complementary DNA (cDNA). Reverse transcription was performed in a T-Personal thermocycler (Biometra, Göttingen, Germany) with a total reaction volume of 15 μl, according to the manufacturer’s instructions. Finally, TaqMan Universal Master Mix II and UNG (Applied Biosystems) and TaqMan fluorescent labeled probes (Applied Biosystems) were used to amplify the cDNA of the studied miRNAs, with a total reaction volume of 20 μl. Specific reaction conditions: UNG activation: 50°C, 2 min; polymerase activation: 95°C, 10 min; 40 cycles: 95°C, 15 s, 60°C, 1 min. The miRNA RT-qPCR primers are shown in Table 1. The *C*_t_ value of the detected sample was analyzed using EcoStudy software v4.0 (Illumina Inc, San Diego, CA, U.S.A.). All samples were standardized by U6 small nuclear RNA (U6 snRNA) as a control. The expression of miRNAs was assessed by a 2^−ΔΔ*C*^_T_ analysis.

**Table 1:** Primers of the miRNA RT-qPCR

miRNAs	Primer sequence
miR-101-3p	F:5′-TGGGCTACAGTACTGTGATA-3′
	R:5′-TGCGTGTCGTGGAGTC-3′
miR-144-3p	F:5′-ACACTCCAGCTGGGTACAGTATAGATGATGTA-3′
	R:5′-CTCAACTGGTGTCGTGGA-3′
miR-153-3p	F:5′-ACACTCCAGCTGGGTTGCATAGTCACAAAAGT-3′
	R:5′-TCAACTGGTGTCGTGGAGTCGGCAATTCAGTTGAGGATCACTTT-3′
miR-185	F:5′-AGGGATTGGAGAGAAAGGCA-3′
	R:5′-AAGGACCAGAGGAAAGCCAG-3′
miR-381-3p	F:5′-ACACTCCAGCTGGGTATACAAGGGCAAGCT-3′
	R: 5′-TGGTGTCGTGGAGTCG-3′
miR-383-5p	F:5′-GTGCAGGGTCCGAGGT-3′
	R:5′-AGATCAGAAGGTGATTGTGGCT-3′
U6	F:5′-ATTGGAACGATACAGAGAAGATT-3′
	R:5′-GGAACGCTTCACGAATTTG-3′

### Transfection

Cells were transfected with 50 nM pre-miR (Applied Biosystems) and 50 ng/cm^2^ pGL3_HSV TK_3′-UTR APP genotype plasmids in HeLa cell lines by Lipofectamine 2000 (Invitrogen, Carlsbad, CA, U.S.A.) .

### Enzyme-linked immunosorbent assay detection

Cells were lysed 24 h after transfection, and total cellular proteins were extracted using the EpiQuik Whole Cell Extraction Kit (Epigentek, Farmingdale, NY, U.S.A.) and the expression of APP protein were detected by the Amyloid Precursor Protein Human ELISA Kit (Life Technologies Corporation, Carlsbad, CA, U.S.A., Catalog # KHB0051), with three replicate wells per sample.

### Genotyping

Genomic DNA was extracted from the blood cells of all subjects, and PCR amplification was carried out using the primers in Table 2. The PCR conditions were: 95°C, 5 min; 94°C, 20 s; 56°C, 30 s; 72°C, 30 s; 35 cycles: 72°C, 5 min; storage at 4°C. After the PCR was completed, the PCR amplification products were purified and sequenced by Shanghai Shenggong Bioengineering Technology Co., Ltd.; the sequencing results were analyzed using Vector NTI 11.5.1 (Invitrogen).

**Table 2:** Primers of APP mRNA RT-qPCR

SNP	Primers sequence
-534G/A	F:5′-GAAATTCCAGGTTGCTCGTG-3′
	R:5′-GGCGTTTCTGGAAGAGAATG-3′
-369C/G	F:5′-CCCCCGCCCCGCAAAATC-3′
	R:5′-TGGGCTTCGTGAACAGTGGGAGGGAGAG-3′
-118C/A	F:5′-ATGATTCAAGCTCACGGGGACGAG-3′
	R:5′-GCTCAGAGCCAGGCGAGTCAGC-3′

### Statistical analysis

Continuous variables were expressed by mean + S.D., and independent-sample *t*tests were used for statistical analyses. Categorical variables were expressed in percentage [*n*(%)] and analyzed using Chi-squared tests. Whether the genotype frequency was consistent with the Hardy–Weinberg equilibrium (HWE) was also assessed with Chi-squared tests. The correlation between SNPs and AD was determined based on the distribution of allele frequencies and genetic models (additive, dominant, and recessive models), and odds ratios (OR) and 95% confidence intervals (CI) were used in an unconditional logistic regression analysis, corrected for age, sex, education, and other factors. SPSS20.0 software (IBM, Chicago, IL, U.S.A.) was used for all statistical analyses. Multivariate dimensionality reduction analysis (MDR) was used to analyze the interaction among -534G/A, -369C/G, and -118C/A loci. All tests were two-tailed, and *P*<0.05 indicated a statistically significant difference.

## Results

### General data

Age, sex, education level, MMSE scores, and clinical dementia rating (CDR) scores of the AD group and the control group are shown in Table 3. There was no significant difference in age, sex, or education (*P*>0.05). The MMSE and CDR scores of the AD group were lower than the scores of the control group (*P*<0.05).

**Table 3:** General data

Parameter	AD (*n*=385)	Control (*n*=385)	*P*
Age (years, mean ± S.D.)	73.6 ± 8.1	72.9 ± 11.3	0.161
Gender [*n*(%)]			0.972
Male	200 (52.0%)	201 (52.2%)	
Female	185 (48.0%)	184 (47.8%)	
Education [*n*(%)]			0.879
Primary school and below	99 (25.7%)	105 (27.3%)	
High school	224 (58.2%)	218 (56.6%)	
University	62 (16.1%)	62 (16.1%)	
MMSE scoring	8.9 ± 3.5	28.6 ± 5.9	<0.001
CDR scoring	2.0 ± 1.0	0.6 ± 0.3	<0.001

### Bioinformatics prediction of miRNA target sites in APP 3′-UTR

To identify new miRNA target sites in human APP 3′-UTR, we used TargetScan (version 7.2) to predict the interaction between miRNAs and APP 3′UTR. The potential targets of miR-101-3p, miR-144-3p, miR-153-3p, miR-185, miR-381-3p, miR-383-5p, and other miRNAs are located in the APP 3′UTR, and the sequences that miRNAs may target are shown in Table 4.

**Table 4:** Human APP 3′UTR miRNAs and target region prediction

Position of APP 3′UTR miRNAs	Predicted pairing of target region (top) and miRNA (bottom)	Seed match*
APP 3′UTR 226-250	5′ UUAAUGGGUU-UUGU-GUACUGUA	
	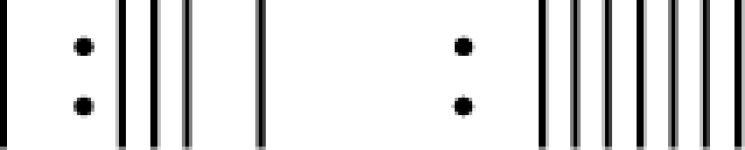	8mer
miR-101-3p	3′ GAAGUCAAUAGUGUCAUGACAU	
APP 3′UTR 514-539	5′ UUUCCAUGACUGCAUUUUACUGUAC	
	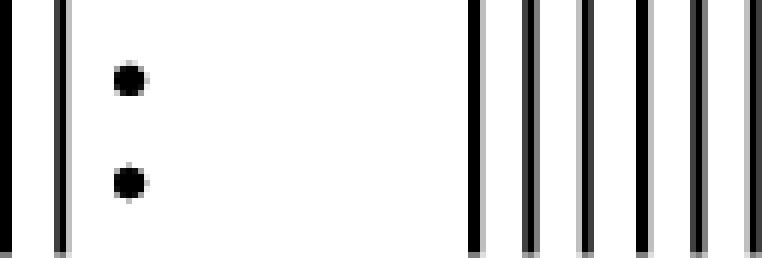	7mer-1A
miR-101-3p	3′ GAAGUCAAUAGUGUCAUGACAU	
APP 3′UTR 219-251	5′ ACUACAUUAUUAAUGGGUUUUGUGUACUGUAA	
	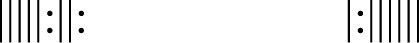	7mer-1A
miR-144-3p	3′ GAUCAUGUAGUAG———–AUAUGACAU	
APP 3′UTR 514-539	5′ …CAUGACUGCAUU—-UUACUGUAC…	
	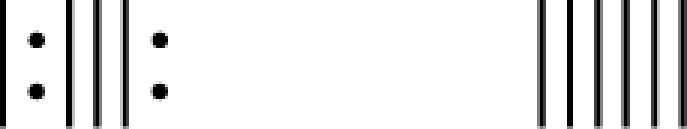	7mer-1A
miR-144-3p	3′ GAUCAUGUAGUAGAUAUGACAU	
APP 3′UTR 440-465	5′ …AUUC-CUUUCCUGAUCACUAUGCAU…	
	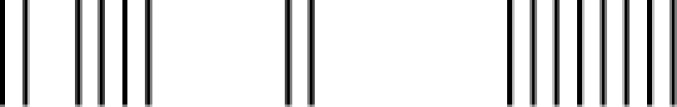	7mer-m8
miR-153-3p	3′ AGUGAAAACAC—UGAUACGUU	
APP 3′UTR 275-295	5′ …AUGAAUAG–AUUCUCUCCU…	
	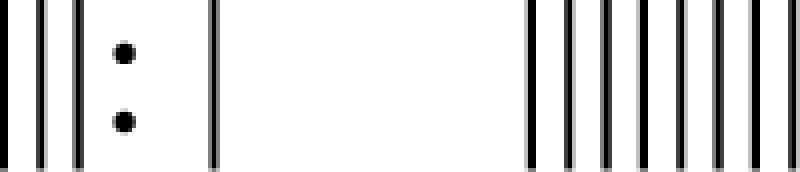	7mer-m8
miR-185	3′ CUUGACGGAAAGAGAGGU	
APP 3′UTR 304-332	5′ …UCACAUAGCCCCUUAGCCAGUUGUAUAU…	
	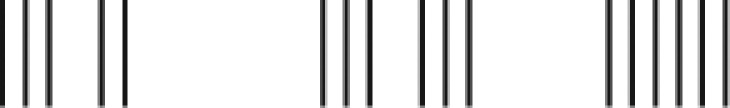	7mer-1A
miR-381-3p	3′ UGUCUC-UCGAA-CGG-GAACAUAU	
APP 3′UTR 430-458	5′ …CUUGCCUAAGUAUUCCUUUCCUGAUCAC…	
	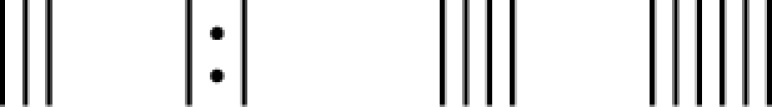	7mer-1A
miR-383-5p	3′ UCGGUGUUA-GUGGAA–GACUAGA	

* 8mer: An exact match with positions 2–8 of the mature miRNA (seed + position 8) followed by an ‘A’. 7mer-m8: An exact match with positions 2–8 of the mature miRNA (seed + position 8). 7mer-A1: An exact match with positions 2–7 of the mature miRNA (seed) followed by an ‘A’.

### Analysis of plasma miRNA expression levels in the AD and the control group

**Figure 1:**
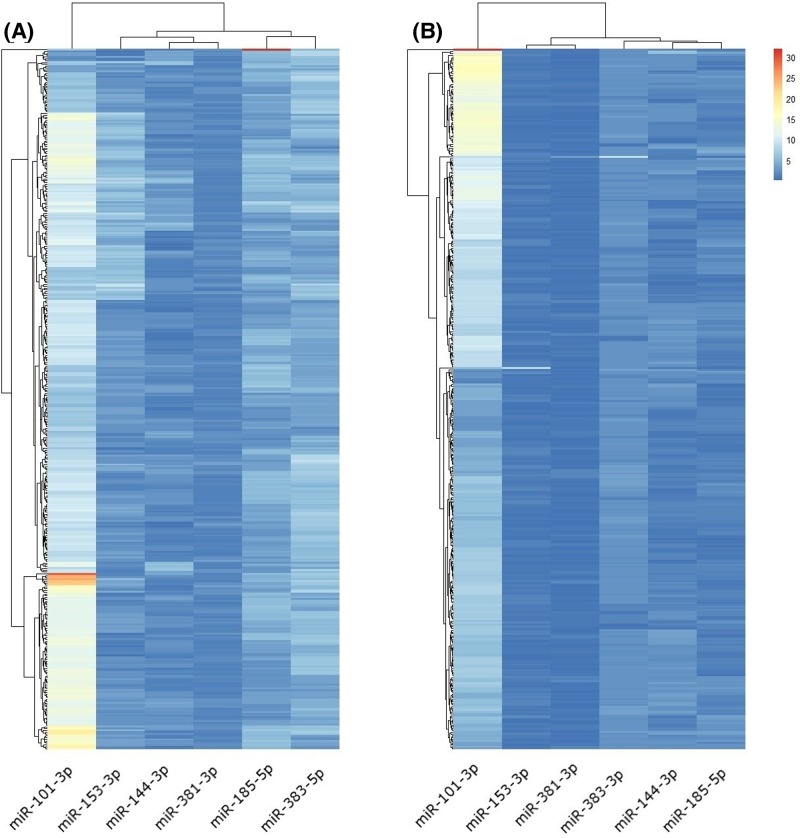
Detection of miRNA expression levels in plasma (**A**) is the expression level of miRNAs in plasma of healthy subjects; (**B**) is the expression level of miRNAs in plasma of patients with AD.

The levels of plasma miRNAs in 385 patients with AD and 385 healthy subjects were detected by RT-qPCR. The results show that the levels of miR-101-3p, miR-153-3p, miR-144-3p, miR-381-3p, and miR-383-5p were significantly lower in patients with AD than in the control group. Differences between groups were statistically significant *P*<0.001, <0.001, <0.001, <0.001, <0.001, <0.001) (Figure 1).

### Association between APP gene polymorphism and AD risk

The genotypic analysis of APP gene-534G/A, -369C/G, and -118C/A loci showed that those three loci genotype frequencies are consistent with the HWE (*P*>0.05). Based on the GG genotype of the APP gene-534G/A locus, GA and AA genotypes were protective factors for AD (adjusted OR = 0.715, 95% CI: 0.571–0.878, *P*=0.001; adjusted OR = 0.540, 95% CI: 0.247–0.957, *P*=0.029) (Table 5). AD risk was lower for the APP gene-534G/A site in the dominant model (adjusted OR = 0.693, 95% CI: 0.559–0.845, *P*<0.001), and there was no AD risk in the recessive model (adjusted OR = 0.576, 95% CI: 0.264–1.019, *P*=0.062); with the G allele as a reference, the A allele was a protective factor for AD risk (adjusted OR = 0.700, 95% CI: 0.573–0.840, *P*<0.001). The risk of AD for CG, GG, and G allele carriers was not significant when compared with the CC genotype as a reference and the C allele at the -369C/G locus of the APP gene (*P*>0.05); in the dominant and the recessive models, AD risk was likewise not significant (*P*>0.05) (Table 5). Taking the APP gene-118C/A site CC allele as a reference, the CA and AA genotypes were protective factors for AD (adjusted OR = 0.781, 95% CI: 0.638–0.941, *P*=0.007; adjusted OR = 0.600, 95% CI: 0.308–0.989, *P*=0.043), the risk of AD decreased in the dominant model (adjusted OR = 0.757, 95% CI: 0.624–0.906, *P*=0.002), and there was no risk of AD in the recessive model (adjusted OR = 0.634, 95% CI: 0.326–1.004, *P*=0.083). Taking the APP gene-118C/A site C allele as a reference, the A allele was a protective factor for AD (adjusted OR = 0.762, 95% CI: 0.639–0.897, *P*=0.001) (Table 5).

**Table 5:** Genotype and allele frequencies of the *APP* -534G/A, -369C/G, and -118C/A loci

	AD (*n*=385)	Control (*n*=385)	*P*	Cruded OR (95% CI)	P*	Adjusted OR (95% CI)
-534G/A						
GG	315 (81.82%)	268 (69.61%)				
GA	63 (16.36%)	100 (25.97%)	<0.001	0.536 (0.370–0.776)	0.001	0.715 (0.571–0.878)
AA	7 (1.82%)	17 (4.42%)	0.017	0.350 (0.130–0.912)	0.029	0.540 (0.247–0.957)
Dominant model			<0.001	0.509 (0.358–0.724)	<0.001	0.693 (0.559–0.845)
Recessive model			0.038	0.401 (0.149–1.039)	0.062	0.576 (0.264–1.019)
G	693 (90.00%)	636 (82.60%)				
A	77 (10.00%)	134 (17.40%)	<0.001	0.527 (0.386–0.720)	<0.001	0.700 (0.573–0.840)
-369C/G						
CC	308 (80.00%)	310 (80.52%)				
CG	65 (16.88%)	67 (17.40%)	0.901	0.976 (0.659–1.447)	0.977	0.988 (0.800–1.191)
GG	12					
(3.12%)	8					
(2.08%)	0.371	1.510 (0.566–4.102)	0.505	1.204 (0.725–1.624)		
Dominant model			0.856	1.033 (0.714–1.497)	0.928	1.016 (0.837–1.208)
Recessive model			0.365	1.516 (0.570–4.107)	0.497	1.206 (0.728–1.624)
C	681 (88.44%)	687 (89.22%)				
G	89 (11.56%)	83 (10.78%)	0.627	1.082 (0.778–1.504)	0.686	1.039 (0.877–1.207)
-118C/A						
CC	299 (77.66%)	259 (67.27%)				
CA	77 (20.00%)	107 (27.79%)	0.006	0.623 (0.439–0.885)	0.007	0.781 (0.638–0.941)
AA	9 (2.34%)					
		19 (4.94%)	0.027	0.410 (0.169–0.976)	0.043	0.600 (0.308–0.989)
Dominant model			0.001	0.591 (0.423–0.825)	0.002	0.757 (0.624–0.906)
Recessive model			0.054	0.461 (0.190–1.091)	0.083	0.634 (0.326–1.004)
C	675 (87.66%)					
	625 (81.17%)					
A	95 (12.34%)					
	145 (18.83%)	<0.001	0.607 (0.454–0.811)	0.001	0.762 (0.639–0.897)	

* Corrected according to general information such as age, sex, body mass index, smoking, and drinking.

### Correlation between APP gene polymorphism and plasma miRNAs levels

**Figure 2:**
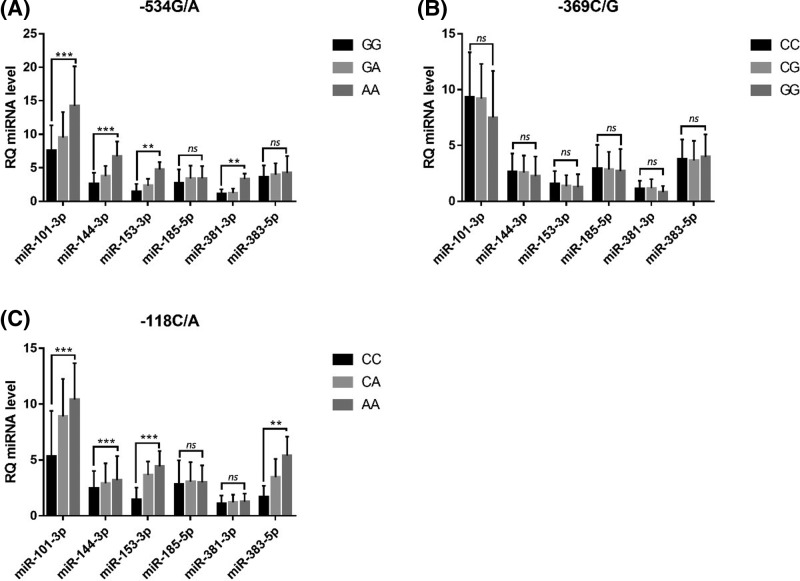
Comparison of plasma miRNAs levels in subjects with different genotypes of APP gene (A) -534G/A, (B) -369C/G, and (C) -118C/A **P<0.01,***P<0.001, ns: not significant.

We analyzed the correlation between plasma miRNAs levels and APP gene polymorphisms. The results showed that plasma miR-101-3p, miR-144-3p, miR-153-3p, and miR-381-3p of subjects with APP-534G/A locus GG, GA, and AA genotypes were elevated (*P*<0.05). There was no statistically significant difference in the level of miR-185 and miR-383-5p between subjects of each genotype (*P*>0.05) (Figure 2A). There were no differences in plasma miR-101-3p, miR-144-3p, miR-153-3p, miR-185, miR-381-3p, and miR-383-5p levels in subjects with CC, CG, and GG genotypes at APP-369C/G locus (*P*>0.05) (Figure 2B). The levels of plasma miR-101-3p, miR-144-3p, miR-153-3p and miR-383-5p in subjects with APP 118C/A locus in CC, CA and AA were increased (*P*<0.05). There were no significant differences in miR-185 and miR-381-3p levels between subjects of each genotype (*P*>0.05) (Figure 2C).

### Analysis of endogenous regulation of APP expression by miRNAs

**Figure 3:**
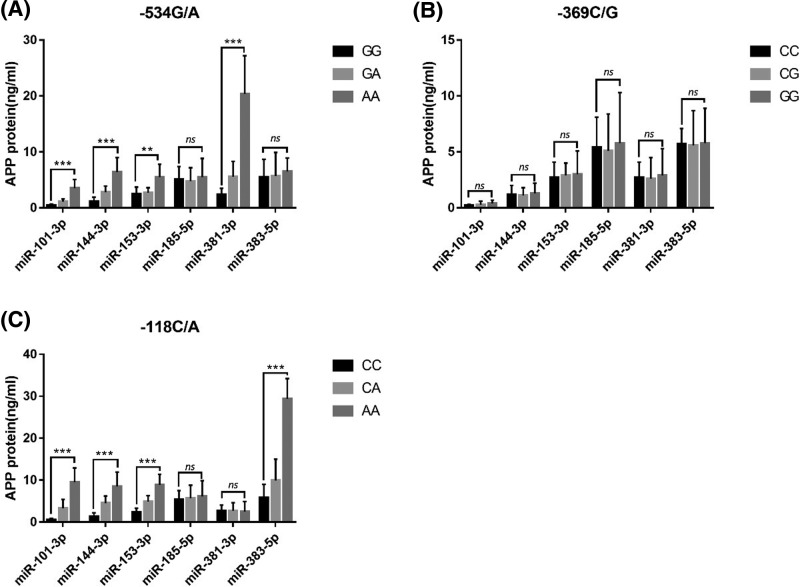
MiRNAs endogenously regulate APP protein expression **P<0.01,***P<0.001.

The results of the enzyme-linked immunosorbent assay (ELISA) kit showed that in HeLa cells transfected with *APP*-534G/A locus GG, GA, and AA genotype plasmids, the highest level of APP protein was detected in the cells transfected with miR-101-3p, miR-144-3p, miR-153-3p, and miR-381-3p, followed by the cells transfected with the GA genotype, and the lowest level detected for the AA genotype (*P*<0.001). There was no difference in APP protein expression among genotypes carrying the APP gene-534G/A locus in the cells transfected with miR-185 and miR-383 (*P*>0.05) (Figure 3A). In HeLa cells transfected with APP-369C/G CC, CG, and GG genotype plasmids, there was no difference in the expression of APP protein in the cells transfected with miR-101, miR-144, miR-153, miR-185, miR-381, and miR-383 (*P*>0.05). (Figure 3B). In HeLa cells transfected with APP-118C/A CC, CA, and AA genotype plasmids, the highest level of APP protein was detected in cells transfected with miR-101-3p, miR-144-3p, miR-153-3p, and miR-381-3p, followed by the CA genotype. The lowest expression was detected for the AA genotype, and the difference was statistically significant (*P*<0.001). However, there was no significant difference in APP protein levels among genotypes carrying the *APP*-118C/A locus (*P*>0.05) detected in the cells transfected with miR-185 and miR-381 (Figure 3C).

### MDR analysis of interaction among SNPs at APP-534G/A, -369C/G, -118C/A loci

**Figure 4:**
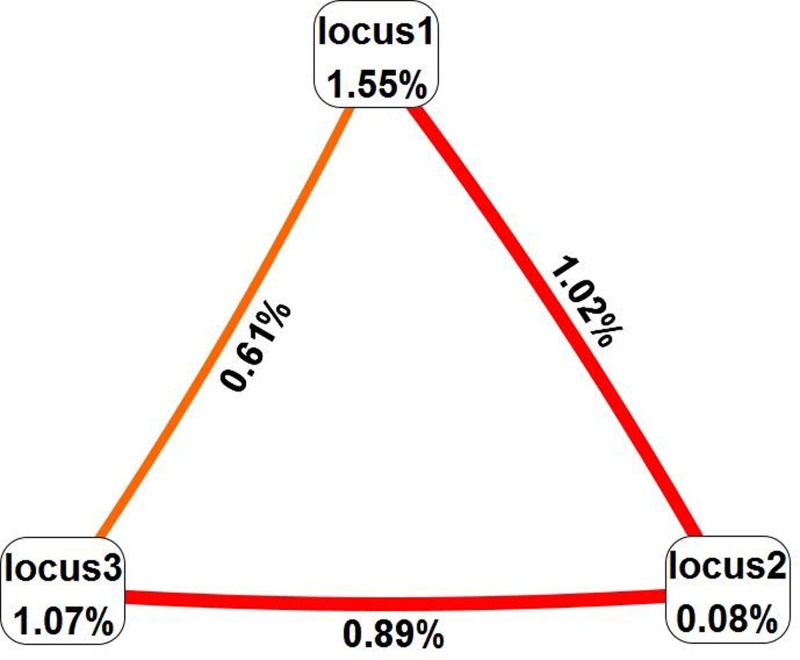
MDR analysis of the interaction between APP-534G/A, -369C/G, and -118C/A site SNPs Locus1 represents the ‘-534G/A’ site, locus2 represents ‘-369C/G’, and locus3 represents the ‘-118C/A’ site. The value at the apex represents the effect on AD, and the larger the value, the greater the risk of AD at the SNP site. The edge values represent interactions between the SNP sites, positive values represent positive interactions, and larger values indicate stronger interactions.

The interaction between SNPs at APP-534G/A, -369C/G, -118C/A loci was analyzed by MDR. The results showed that the interaction between-534G/A locus and-369C/G locus was the strongest, the interaction between-369C/G, -118C/A locus was the second, and the interaction between-534G/A locus and-118C/A locus was the smallest (Figure 4).

### Hierarchical analysis of miRNA levels and age, gender, and MMSE

**Figure 5:**
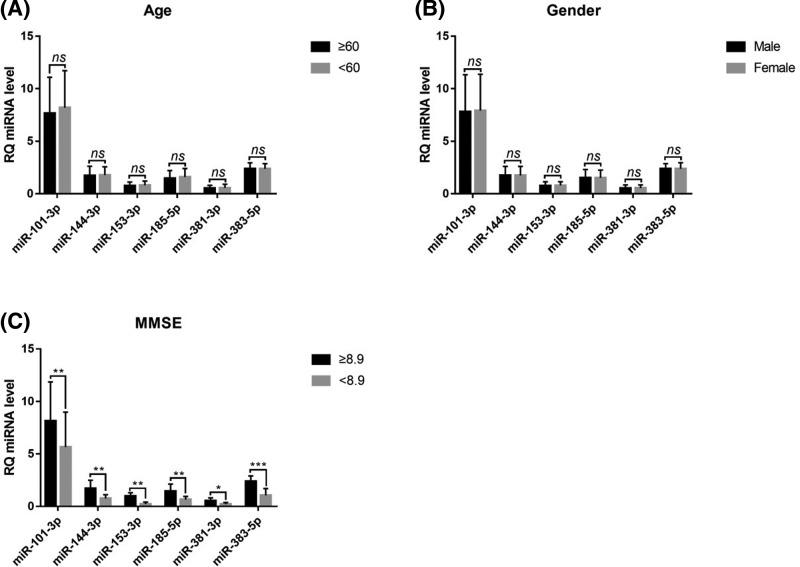
Comparison of miRNA levels in patients of different ages, genders, or MMSE AD *P<0.05, **P<0.01, ***P<0.001, ns: not significant.

A comparative analysis of plasma miRNAs levels in AD patients with different ages, genders, and MMSE revealed that there were no differences in levels of miRNAs between AD patients of different ages (*P*>0.05), including miR-101-3p, miR-153-3p, miR-144-3p, miR-381-3p, miR-185-5p and miR-383-5p (*P*>0.05) (Figure 5A). There was no difference in plasma miR-101-3p, miR-153-3p, miR-144-3p, miR-381-3p, miR-185-5p, miR-383-5p levels between male and female AD patients (*P*>0.05) (Figure 5B). Plasma levels of miR-101-3p, miR-153-3p, miR-144-3p, miR-381-3p, miR-185-5p, and miR-383-5p were significantly higher when MMSE ≥ mean (8.9). AD patients with MMSE < mean (8.9) (*P*<0.05) (Figure 5C).

## Discussion

In recent years, the molecular mechanism and early diagnosis of AD occurrence and development have become the focus of research, especially the biomarkers for specific early warning signs [27,28]. Studies have found that free miRNAs in plasma can be used as biomarkers for a variety of diseases, such as Parkinson’s disease [29], tumors [30], and AD [31]. Some researchers have found that miRNAs in peripheral blood play an important role in the diagnosis of AD [19–21]. The current study shows that the levels of free miR-101-3p, miR-153-3p, miR-144-3p, miR-381-3p, and miR-383-5p in the plasma of patients with AD are lower than the levels in healthy subjects, making them potential molecular markers of AD. In addition, bioinformatics research based on target prediction found that all of these miRNAs target the APP 3′UTR region; whether mutations in this region affect the efficiency of miRNAs targeting regulation is a key issue worthy of study.

AD is one of the most common forms of dementia, and its neuropathological markers include Aβ and τ accumulation, which manifest as senile plaques and neurofibrillary tangles or dystrophic neuritis [32]. Aβ is derived from its parental molecule Aβ precursor protein (APP), which is usually cleaved by β-secretase (BACE1) from the Aβ N-terminus to form soluble Aβ [33]. The human APP gene is located on chromosome 21, and alternative splicing produces 8–11 kinds of APP protein isoforms with different protein amino acid lengths [34]. APP overexpression is closely related to the occurrence of AD [35], and can therefore be used to reduce the production of AD by repressing the expression of APP and Aβ in sporadic AD. APP 3′UTR is a key region for miRNAs to target the regulation of APP expression. According to earlier research, genetic polymorphisms of this site are related to the efficiency of miRNAs targeting regulation [36]. Using bioinformatics to predict miRNAs targeting the expression of the APP gene, we found six kinds of miRNAs, namely miR-101-3p, miR-153-3p, miR-144-3p, miR-381-3p, and miR-383-5p. Studies have shown that miR-101-3p markedly reduces the expression of reporter genes under the control of APP 3′-UTR in human cell culture [37]. Specific target protectants that block the interaction between miR-101-3p and its functional target site within the APP 3′-UTR enhanced the level of APP expression in HeLa. Thus, endogenous miR-101-3p regulates the expression of APP in human cells through specific sites located within its 3′-UTR. The results of the present study show that miR-101-3p is down-regulated in AD, consistent with the results of Hebert et al. [38]. miR-153-3p is associated with the development of many human diseases, such as non-small cell lung cancer [39], liver cancer [40], and others. The initial detection of miR-153-3p involvement in neurodegenerative diseases has been associated with α-synuclein regulation in PD, a protein that is also involved in both familial and sporadic AD pathology. The results of the current study show that the expression of miR-153-3p is also down-regulated in patients with AD, consistent with the results of Junn et al. [41]. The *miR-144-3p* gene, located on chromosome 10, was originally identified as an anti-cancer gene and plays a crucial role in tumor cell proliferation, apoptosis, invasion, and metastasis [42]. Up-regulation of miR-144-3p expression is often accompanied by persistent cognitive decline [43]. miR-144-3p can also be targeted to bind to APP 3′UTR, and studies have shown that miR-144-3p overexpression markedly inhibits APP protein expression [44], consistent with the results of the current study. miR-381-3p is located in a cluster within the 14q32.31 chromosomal region. It has been shown that miR-381-3p is associated with the development of various diseases, and its down-regulation can inhibit lung cancer [45], breast cancer [46], and colorectal cancer [47]. miR-381-3p is therefore considered to be a potential tumor suppressor. miR-383-5p is also a disease-associated miRNA, and its ectopic expression has been shown to be involved in cell growth, apoptosis, and expression of apoptosis-related proteins [48]. At present, although bioinformatics are used to predict the binding of miR-381-3p and miR-383-5p to APP 3′UTR, there are only few studies on the correlations between miR-381-3p, miR-383-5p, and AD. The results of the current study show that the levels of miR-381-3p and miR-383-5p in the plasma of patients with AD are down-regulated. We believe that these decreased expression levels of miR-381-3p and miR-383-5p may lead to an increase in APP expression and soluble Aβ levels and to the onset of AD; further research into this issue is however needed.

At the same time, we also found that genetic variations of the -534G/A and -118C/A sites in the APP 3′UTR region affected the binding of miRNAs to APP 3′UTR and thus the regulation of APP expression by miRNAs. The -534G/A locus variation affects the binding of miR-101-3p, miR-144-3p, miR-153-3p, miR-381-3p, and APP 3′UTR. The -534G/A locus A allele is a protective factor for AD, but in the present study, the APP protein level in transfected A allele cells was significantly higher than the G allele. We speculate that there may be multiple miRNAs involved in the regulation of APP protein expression *in vivo*. The co-regulation of multiple miRNAs in individuals carrying the A allele down-regulates APP expression. When miR-101-3p, miR-144-3p, miR-153-3p, and miR-381-3p were transfected alone, APP expression was up-regulated. The specific mechanism needs further research.While the -118C/A site affects miR-101-3p, miR-144-3p, miR-153-3p, and miR-383-5p binding to APP 3′UTR. Similarly, the -118C/A site A allele is a protective factor for AD. However, APP expression in cells of the A allele was increased after transfection of miR-101-3p, miR-144-3p, miR-153-3p and miR-383-5p, respectively. We believe that there may be other miRNAs involved in the regulation of APP expression in the body, and the specific mechanism needs further study. In addition, the results of the current study suggest that the genetic variation at the -369C/G locus is unrelated to the occurrence of AD, and that the regulatory effect of miRNAs on APP protein expression levels is not related to the genetic variation at the -369C/G locus. Although Theuns et al. found that the -369C/G locus is associated with APP transcriptional activity, which is inconsistent with the results of the current study, we believe that this may be related to inconsistencies in the genetic background of different ethnic groups.

The present study has several shortcomings that need to be mentioned. First, due to limitations of the sample collection, we could not obtain AD brain samples to analyze the correlation between APP expression levels and miRNAs and the relationships between different genetic variations of APP 3′UTR. Second, the number of rare mutation carriers is small, which may have affected the statistical analyses.

## Conclusion

Genetic polymorphisms of the APP 3′UTR-534G/A and APP-118C/A loci can affect the development of AD through the regulation of APP expression by miRNAs. Genetic polymorphisms may thus affect the usability of miRNAs as potential targets for AD diagnosis.

## Abbreviations

AD: Alzheimer’s disease
APP: amyloid precursor protein
Aβ: amyloid-β
BACE1: β-secretase
cDNA: complementary DNA
CDR: clinical dementia rating
95% CI: 95% confidence interval
CXC: chemokine receptor
HWE: Hardy–Weinberg equilibrium
MDR: multivariate dimensionality reduction analysis
miRNA: microRNA
MMSE: Mini-Mental State Examination
OR: odds ratio
PD: progressive disease
Rt-qPCR: quantitative real-time polymerase chain reaction
SNP: single nucleotide polymorphism
UTR: untranslated region
